# Stigma, violence and HIV vulnerability among transgender persons in sex work in Maharashtra, India

**DOI:** 10.1080/13691058.2016.1271141

**Published:** 2017-01-30

**Authors:** Deepika Ganju, Niranjan Saggurti

**Affiliations:** HIV and AIDS Program, Population Council, New Delhi, India

**Keywords:** Transgender sex workers, India, stigma, violence, HIV, community mobilisation

## Abstract

Among marginalised groups in India, HIV prevalence is highest among transgender persons; however, little is known about their HIV vulnerability. This study describes transgender sex workers’ experiences of stigma and violence, a key driver of the HIV epidemic, and explores their coping responses. In-depth interviews were conducted with 68 respondents in Maharashtra state, India. Findings show that respondents face pervasive stigma and violence due to multiple marginalised social identities (transgender status, sex work, gender non-conformity), which reinforce and intersect with social inequities (economic and housing insecurity, employment discrimination, poverty), fuelling HIV vulnerability at the micro, meso and macro levels. Several factors, such as felt and internalised stigma associated with psycho-social distress and low self-efficacy to challenge abuse and negotiate condom use; clients’ power in sexual transactions; establishing trust in regular partnerships through condomless sex; norms condoning violence against gender non-conforming persons; lack of community support; police harassment; health provider discrimination and the sex work environment create a context for HIV vulnerability. In the face of such adversity, respondents adopt coping strategies to shift power relations and mobilise against abuse. Community mobilisation interventions, as discussed in the paper, offer a promising vulnerability reduction strategy to safeguard transgender sex workers’ rights and reduce HIV vulnerability.

## Résumé

Des populations marginalisées en Inde, les personnes transgenres sont celles parmi lesquelles la prévalence du VIH est la plus élevée ; cependant, les connaissances restent limitées sur leur vulnérabilité au VIH. Cette étude décrit les expériences de stigmatisation et de violence vécues par des travailleur(se)s du sexe transgenres, un moteur essentiel de l’épidémie de VIH, et explore leurs stratégies d’adaptation. Des entretiens ont été conduits avec 68 répondant(e)s dans l’État du Maharashtra, en Inde. Les résultats montrent que les répondant(e)s sont confronté(e)s à une stigmatisation et à une violence, omniprésentes en raison de multiples identités sociales marginalisées (statut de transgenre, travail du sexe, non-conformité vis-à-vis du genre), ce qui renforce et recoupe les inégalités sociales (insécurité économique et sur le plan du logement, discrimination à l’embauche, pauvreté) qui elles-mêmes alimentent la vulnérabilité au VIH aux niveaux micro, méso et macro. Plusieurs facteurs, tels le stigma ressenti et intériorisé, associé à la détresse psychosociale et à une faible auto-efficacité dans la résistance aux abus et la négociation du préservatif ; le pouvoir des clients dans les transactions sexuelles ; l’installation de la confiance dans les partenariats réguliers à travers les rapports sexuels sans préservatifs ; les normes qui excusent la violence à l’endroit des personnes ne se conformant pas au genre ; l’absence de soutien communautaire ; le harcèlement de la police ; la discrimination exercée par les prestataires de santé et l’environnement du travail du sexe, créent un contexte pour la vulnérabilité au VIH. Face à une telle adversité, les répondant(e)s adoptent des stratégies d’adaptation pour changer les rapports de force et se mobilisent contre les abus. Les interventions de mobilisation communautaire constituent une stratégie prometteuse de réduction de la vulnérabilité pour protéger les droits des travailleur(se)s du sexe transgenres et diminuer leur vulnérabilité au VIH.

## Resumen

Entre los grupos marginados de la India, la prevalencia más alta del virus del sida se encuentra entre personas transexuales, sin embargo, se conoce poco su vulnerabilidad con respecto al VIH. En este estudio describimos qué experiencias tienen los transexuales que comercian con el sexo con respecto al estigma y la violencia, que son factores importantes en la epidemia del sida, y analizamos los mecanismos de defensa que utilizan. Para este estudio se llevaron a cabo entrevistas exhaustivas con 68 participantes en el estado de Maharashtra, India. Los resultados indican que los entrevistados se enfrentan a estigma y violencia generalizados debido a las diversas identidades sociales marginadas (estado transexual, trabajo sexual, disconformidad con su sexo) que solapan y refuerzan las desigualdades sociales (inseguridad económica y precariedad de la vivienda, discriminación laboral, pobreza), incentivando la vulnerabilidad al VIH en niveles micro, meso y macro. Existen varios factores que crean un contexto para la vulnerabilidad al VIH, tales como el estigma sentido e interiorizado asociado a angustia psicosocial y un nivel bajo de autoeficacia para enfrentarse al abuso y la negociación del uso de preservativos; el poder de los clientes en las transacciones sexuales; generar confianza en las relaciones regulares mediante las relaciones sexuales sin preservativos; normas que condonan la violencia contra personas no conformes con su sexo; la falta de apoyo comunitario; el acoso policial; la discriminación por parte de profesionales de la salud y el entorno del trabajo sexual. Frente a tal adversidad, los entrevistados utilizan mecanismos de defensa para cambiar las relaciones de poder y movilizarse contra los abusos. Las intervenciones de movilización en la comunidad, tal como se describen en el artículo, suponen una estrategia prometedora para reducir la vulnerabilidad y proteger los derechos de los transexuales que comercian con el sexo, y también para disminuir la vulnerabilidad al VIH.

## Introduction

Transgender persons, whose gender identity or expression differs from their biological sex, are amongst the population groups most affected by HIV. Worldwide, transgender persons are 49 times more likely to be HIV infected than the general adult population (Baral et al. 2013); and transgender persons who enage in sex work are far more vulnerable to HIV (Operario, Soma, and Underhill 2008). In India, the estimated transgender HIV prevalence (8.82%) is about 20 times higher than the general population, and highest among key populations (female sex workers, injecting drug users and men who have sex with men (NACO 2014a).

Transgender persons’ HIV vulnerability has been linked to violence and stigma (Wilson et al. 2011). Physical and sexual violence is associated with inconsistent condom use, and increased HIV/sexually transmitted infection (STI) vulnerability among sex workers (Beattie et al. 2010), while stigma is linked to adverse mental and physical health outcomes, and housing, healthcare and employment inequities among transgender persons (White Hughto, Reisner, and Pachankis 2015). Stigma is a process of labelling, stereotyping, devaluing and discriminating within unequal power structures, based on actual or perceived identity or status (Link and Phelan 2006), and manifested in loss of status, unfair treatment and isolation (Poteat et al. 2015; White Hughto, Reisner, and Pachankis 2015). Among transgender persons and other marginalised communities, multiple stigmas intersect with socio-structural inequities (Logie et al. 2011), increasing vulnerability to violence, HIV and negative health outcomes (Baral et al. 2013; Bockting et al. 2013; Poteat et al. 2015; Sugano, Nemoto, and Operario 2006).

In India, transgender persons experience elevated HIV vulnerability due to high levels of violence and stigma (Chakrapani et al. 2011; Newman et al. 2008; Setia et al. 2006; Shaw et al. 2012). A significant proportion of transgender persons in India engage in transactional sex (Newman et al. 2008; Setia et al. 2006; Shaw et al. 2012). As documented, sex work increases vulnerability to physical and sexual violence (Newman et al. 2008; Shaw et al. 2012), which is linked to the risk of HIV transmission (Poteat et al. 2015): coerced sex is rarely protected (Deering et al. 2013), is a barrier to condom negotiation (Panchanadeswaran et al. 2008) and increases the likelihood of condom failure (Choi, Chen, and Jiang 2008). Transgender persons are often mobile for sex work, elevating their vulnerability to violence and HIV (Ramesh et al. 2012).

Although sex workers, particularly transgender sex workers, are frequently exposed to stigma and violence, they have limited recourse to challenge abuse (Poteat et al. 2015). Within coercive and stigmatising environments, sex workers also adopt coping strategies and use social support and other resources (Logie and Daniel 2016). Studies also highlight the strengths of community mobilisation interventions among sex workers in addressing structural risks, including stigma and violence (Ghose et al. 2008).

While mobile transgender sex workers are arguably amongst the groups most vulnerable to HIV, most studies in India have explored the HIV vulnerability of men who have sex with men and transgender persons together, with limited evidence on how stigma and violence impact transgender sex workers’ disproportionate HIV vulnerability in India. Given this information gap, our study explores transgender sex workers’ experiences of stigma and violence in Maharashtra, a high HIV prevalence state in India with a large transgender population. Specifically, the study explores how respondents’ experience stigma and violence in the context of HIV vulnerability and the strategies they adopt to address stigma and violence. An understanding of these experiences can inform HIV prevention interventions for transgender persons in India.

We used a modified social ecological framework to guide our understanding of transgender sex workers’ experiences of stigma – felt stigma (shame/fear of rejection), internalised stigma (acceptance of negative feelings) and symbolic stigma (othering, blaming and shaming of marginalised groups/associated people) (Herek 2007; Herek, Capitanio, and Widaman 2003) – violence, including rejection, discrimination, physical and sexual violence and HV vulnerability; and respondents’ coping strategies. These were explored across the micro (individual, interpersonal), meso (community) and macro (institutional, structural) levels (Logie et al. 2011). The model was applied after completed analyses to organise themes and show connections between experiences of stigma, violence, HIV vulnerability and coping.

## Transgender persons in India

Although transgender persons have always been victims of stigma and violence in India due to their transgressive gender identity (Reddy 2005), they were traditionally believed to have the power to confer fertility, giving them socio-religious legitimacy. Their traditional occupations of begging and dancing at weddings are now declining with increased urbanisation and changing social structures, and many have become sex workers in towns and cities (Sahastrabuddhe et al. 2012; Setia et al. 2006). Transgender persons are generally the receptive partners in sexual partnerships (Reddy 2005). Recently, transgender persons were granted legal status in India.

Transgender persons often migrate from their home village to cities to escape restrictive families, to find peer support and for better earning opportunities (Setia et al. 2006). They generally belong to a hierarchal clan or *gharana* (meaning house); each *gharana* is headed by a *guru* (leader), who has several disciples under him. G*urus* closely control their disciples’ daily lives and provide mentorship on community customs and rituals (Kalra 2012).

Until recently, HIV prevention programmes in India included transgender persons under the broader category of ‘men who have sex with men and transgender persons’. The Government of India’s National AIDS Control Programme IV (2012–17) recognises transgender persons as a separate group for programme focus (NACO 2014b) and since 2009, Avahan, the India AIDS initiative, has rolled out a community mobilisation programme to reduce vulnerability among marginalised groups – female sex workers, men who have sex with men and transgender persons– in high HIV prevalence states.

## Methods

This paper uses information from qualitative research drawn from in-depth interviews with 68 self-identified transgender persons (locally called *hijras*) engaged in sex work in Maharashtra, India. The study objectives were to explore the profile and mobility patterns of transgender sex workers, determinants of HIV vulnerability and available support systems. This is part of a larger mixed-methods study in four high HIV prevalence states of India – Andhra Pradesh, Maharashtra, Tamil Nadu and Karnataka– designed to understand patterns, drivers and determinants of mobility and linkages with HIV vulnerability among sex workers (female sex workers and transgender sex workers) and male migrant workers (Saggurti et al. 2012).

To ensure the study was relevant to transgender sex workers’ issues and needs, members of the transgender sex worker community from the state HIV prevention programme were involved in the research process, including training interviewers on sensitive issues, providing feedback on the study guide, identifying areas where community members congregate and recruiting respondents. They also assisted in interpretation of initial results, and have been engaged with follow-up intervention activities, which were part of the HIV prevention programme implemented in the state.

### Study sample

Respondents were selected from five districts of Maharashtra – Mumbai, Pune, Nashik, Ahmednagar and Thane – representing the highest concentration of transgender persons and the highest HIV prevalence (based on mapping and sentinel surveillance data, respectively) (NACO 2007). Three or four sites were purposively selected in each district (totalling 16) through discussions with local non-governmental organisation (NGO) programme managers working with transgender sex worker communities, health providers and health centre/NGO outreach workers. Thereafter the field team contacted individuals in each site who were knowledgeable about the local transgender community – brokers/agents for transgender sex workers, community leaders, *guru*s*,* rickshaw drivers and sex workers themselves – to identify areas where communities live, engage in sex work and solicit clients. This process also helped to build community rapport and identify participants for recruitment in each site.

As transgender sex workers are a highly marginalised group, we used a combination of purposive and snowball sampling methods to recruit participants. Recruitment was facilitated by partnerships with local NGOs working in the study sites, through their network of outreach workers and peer educators. Additionally, respondents were asked at the close of the interview to identify others who could be recruited. Inclusion criteria were: self-identification as male-to-female transgender (classified as male at birth but identifying as female); 18 years or older; migrated from the home village and engaged in sex work in the destination area.

### Data collection

A semi-structured interview guide with open-ended questions was used to obtain detailed narratives of the context and circumstances surrounding stigma, violence and HIV vulnerability. Key topics explored were transitioning as a transgender person, entry into sex work, migration history, mobility for sex work, sexual partnerships (regular/paid), HIV risk behaviours (condomless sex, multi-partner sex), condom negotiation, family relations, stigma and violence, support systems and health seeking.

Interviews were conducted by a team of 10 investigators fluent in English and Marathi (local language), with a post-graduate degree and experience in qualitative research with transgender populations. Prior to data collection, the team underwent in-depth training on rapport building, interviewing techniques and use of the interview guide. Interviews were conducted in private locations that ensured confidentiality and were convenient to respondents. Each interview lasted approximately 75–90 minutes. Interviews were conducted in Marathi, audio-taped after obtaining permission, transcribed, translated and typed into English by the interviewer and saved as Word files for subsequent analysis. To ensure translation accuracy, each translated interview was checked with the audiotape for transcription errors by an investigator who had not conducted the interview, and queries resolved through discussion within the research team.

Procedures for this study were reviewed and approved by the Population Council’s Institutional Review Board (protocol #390). Verbal consent was obtained from all respondents prior to interview. No personal identifiers were collected. Participants were not given any monetary compensation but were referred to local organisations for services if required.

### Data analysis

Transcripts were analysed using thematic analysis (Braun and Clarke 2006). All textual data were analysed by the authors using an inductive and iterative process. The initial coding framework was based on key themes reflected in the interview guide (e.g. experiences of stigma and violence, social support), following which conceptually driven substantive codes (e.g. self-efficacy, psycho-social distress, context of sex work) were applied. Contradictions within and between themes and sub-themes were resolved through an iterative process (Strauss and Corbin 1990). Inter-coder reliability was achieved by having both authors independently code the same 10 interviews, based on identified themes and codes related to how respondents experienced and coped with stigma and violence over their life course. Data were coded using Atlas Ti 5.0, and the coded data were used to answer the key research questions in this paper. Participants were given fictitious names to ensure confidentiality.

## Findings

Respondents were aged 20–40 years; overall 78% (53/68) were ≤ 30 years. A total of 34% (23/68) had no education, 25% (17/68) had ≤ 5 years of schooling, 32% (23/68) had 6–10 years of schooling and just 5 had a higher education. Some respondents were living in *gharanas* in the destination area, while others were living on their own or with a few peers.

Respondents’ experiences of stigma and violence spanned the micro, meso and macro levels, creating a context for HIV acquisition and transmission.

### Micro level: stigma, violence and HIV vulnerability

Micro experiences of felt and internalised stigma were associated with psycho-social distress and low self-efficacy to challenge abuse and negotiate condom use.

#### Intrapersonal factors

##### Psycho-social distress

Respondents commonly described feelings of intense shame and humiliation (felt stigma) linked to social isolation and physical violence. Felt stigma was associated with ‘coming out’ visibly as non-gender identifying males – dressing and behaving as girls – in childhood. Stigma, violence and lack of family support had psychological consequences, including low self-esteem, depression and suicidal ideation, and many had run away from abusive homes:

Neighbourhood boys would tease me. My brother would say, ‘Why do you behave like girls? Don’t come to work, we will lose our prestige.’ I became very unhappy. I told the transgender persons who had come to our village, ‘I don’t want to live, I want to die’. (Poonam, 24 years, university graduate)

Respondents described low self-worth related to being a transgender person and sex worker and their inability to meet the normative roles of ‘marriage’ and procreation, which were linked to partnership instability:

Who [which sex partner] will come regularly? If he comes once he doesn’t come again. What will he get from me that he will come again? Every time new clients come. … I loved a man but I left him. What could I do? He wanted to marry and have children, so I left him*.* (Revati, 38 years, no education)

##### Low self-efficacy to challenge abuse

Study participants had internalised negative community attitudes (internalised stigma) towards themselves. As the following quotation shows, acceptance of negative family attitudes continued even after leaving home, undermining the ability to seek family support:

I have severed family relations. If I go to the village and meet them they will feel ashamed and everyone in the village will laugh at me and my family*.* (Punita*,* 23 years, completed 7 years of schooling)

Felt and internalised stigma appear to reinforce study participants’ perceptions that they must expect and accept stigma and violence in community interactions. In client interactions this resulted in low self-efficacy to challenge abuse and negotiate condom use:

A client wanted oral sex and I said I do not like it. He started forcing me. Then he beat me. I did not do anything. What can a transgender person do to him? (Radha, 34 years, completed 7 years of schooling)

‘Rowdies’ [an Indian term for hooligans] keep teasing us, abusing us and beating us. They force us to have sex without a condom … whenever we see them we run away. (Vimla, 29 years, completed 10 years of schooling)

For devalued respondents, low self-worth and the need for intimacy in regular partnerships presented a barrier to condom use:

I have sex with my ‘husband’. I am not married but I consider him my husband. I don’t use a condom with him because other than me, he doesn’t have sex with anyone. I have full faith that he will not go [have sex] to anyone else. (Smita, 21 years, completed 10 years of schooling)

Similarly, respondents’ acceptance that the police had the right to perpetrate violence made it difficult to resist abuse:

When I complained to the police about being raped, they said, ‘They are hooligans … where to look for them?’ I did not go back because I know the police will not take any action. (Reena, 21 years, completed 10 years of schooling)

Discrimination in health settings was also internalised; as the following quote illustrates, study participants excluded themselves from the category of ‘good’ people who are treated respectfully:

Many doctors leave us and treat ‘good’ men and women first. They give us treatment right at the end. (Lakshmi, 38 years, no education)

Respondents appeared to accept that their gender non-conformity justifies their exclusion from non-traditional forms of employment:

How long can we keep begging? Sometimes people give us money and sometimes they don’t. To earn money we have to do something. And nobody gives other work to transgender people. So I started doing this [sex work]. (Sunita, 23 years, completed 4 years of schooling)

#### Interpersonal factors

Forced sex, condomless sex, homelessness, rejection and social isolation perpetrated by sexual partners, family and peers characterised daily interactions.

##### Clients

A commonly reported theme was pervasive client violence: ‘Clients come anytime to beat me’. Violence was enacted with impunity, as transgender sex workers were perceived to be sexual objects whose rights did not need to be respected*.* Clients exercised control over sexual transactions, including type of sex act, payment terms and condom use, and sometimes even refused to pay for services:

Everywhere there are fights with clients. It is about money; sometimes condomless sex. (Beena, 26 years, completed 7 years of schooling)

It all depends on the client. If he wants to use it [condom] then we do, [or] else, no. I don’t insist. (Smita, 21 years, completed 10 years of schooling)

Transphobia-related violence was described; clients often reacted violently when they found that the ‘woman’ sex worker was a transgender person:

Clients come to us thinking we are women. When they find out we are transgender persons, they abuse and fight. Some come knowing we are transgender persons, but after sex they say they did not enjoy it, and ask for their money back. (Vinita, 28 years, completed 2 years of schooling)

##### Regular partners

Steady partnerships were associated with being cheated, beaten and forcibly evicted from home, compounding respondents’ economic and housing insecurity. Due to stigma associated with sex work and transgender status, regular partners pressurised some participants to give up sex work and avoid their peers:

I loved him [regular partner]. I stopped doing sex work because he did not like it. He did not like me meeting my transgender peers, so I would secretly meet them. He would scold me and beat me. Once I went to see a film with a peer and he saw me. He scolded me a lot, beat me and drove me out of his house. (Madhu, 32 years, no education)

##### Family

Family enacted stigma and violence – being isolated, beaten and taunted – when respondents transitioned to becoming a transgender in childhood were described. Family rejection was commonly reported due to the fear of being ‘shamed’ (symbolic stigma). In some cases, respondents were forcibly evicted from home:

People would not stop taunting my parents. My parents kept me forcibly at home because they were afraid everybody would know their son is like this. I told them that if they did not let me go out I would run away. But they did not understand my feelings. (Meena, 23 years, completed 3 years of schooling)

##### Peers

Narratives describe peer intimidation and physical violence over clients and ‘solicitation’ territory, or for deviating from *gharana* rules, resulting in loss of emotional and social support, and livelihood and housing insecurity:

Our *guru* was very hot-tempered. We did not tell him about our sex work activities. When he got to know, he beat me and said, ‘Don’t show your face again’ and drove me out of the house. (Vimla, 30 years, completed 10 years of schooling)

### Meso level: stigma, violence and HIV vulnerability

Stigmatising norms that devalue sexual minorities and shape community behaviours perpetuate stigma, exclusion, discrimination and violence against transgender sex workers.

#### Norms

In India, where marriage and procreation are regarded as key criteria to achieve respect, norms appear to justify stigma and violence against gender non-conforming groups. The following quotation illustrates community concerns about transgressing gender norms:

This boy will not be happy with us. He can only be happy with the transgender peer group. We cannot get him married. Even if he gets married, he will be unhappy. It’s better to send him to the *guru*. (Anita, 23 years, completed 7 years of schooling)

#### Community members

Norms devaluing transgender sex workers, related to visible gender differences and street-based activities (begging and sex work), condone stigma and violence in daily interactions. Symbolic stigma (blaming and shaming) and violence were pervasive*.* Respondents described social isolation, verbal and physical harassment, and having to leave their homes to ensure safety:

I had a fight with the neighbourhood boys. They beat me saying, ‘Why do you stand here? Don’t stand here.’ Then they started abusing me, and everyone nearby started beating me. After some days there was a fight. Some people slapped and abused us, and the neighbours looked at us with contempt. In many places such violence takes place. (Alia, 20 years, completed 10 years of schooling)

Male rowdies were reported as frequently perpetrating brutal violence. Respondents recounted experiences of forcible abduction from public spaces and gang rape. Narratives suggest that rowdies feel a sense of entitlement to intimidate and harass respondents and are able to do so without being challenged in settings where sex workers are perceived to be ‘easy game’ to be sexually exploited:

Hooligans harass us and nobody dares oppose them. They raped my transgender friend. She was too scared to tell anyone. She was taken by force in front of the shopkeepers. Nobody dared say anything. (Meera, 23 years, completed 8 years of schooling)

Lack of community support was also a barrier to addressing abuse:

If someone beats us, everyone watches. Nobody dares to help. We have to run away. (Ila, 32 years, no schooling)

### Macro level: stigma, violence and HIV vulnerability

Macro-level factors elevated vulnerability to forced sex, condomless sex and compromised care.

#### Police

The police, who have daily contact with street-based sex workers, posed the greatest threat to respondents’ physical and sexual safety. Multiple instances of threatened or enacted police violence to obtain free sexual services, as well as arbitrary arrest and beatings, were described. In the context of the criminalisation of same-sex relations, the police appeared to feel a sense of entitlement to engage in coerced sex:

The police give us maximum trouble. They beat us and we run away. If they catch us we have to pay a lot of money … whatever money we have they take away. They [police] try to have sex with us for free, that too without a condom. If we do not agree they beat us and we have to pay money to get released*.* (Vimla, 29 years, completed 10 years of schooling)

Ongoing police harassment resulted in sex workers being forced to relocate, or work in unfamiliar locations, elevating vulnerability to abuse:

The police were giving us a lot of trouble. Often I had to pay them to get released so I moved from there. (Rima, 37 years, completed 5 years of schooling)

#### Health providers

Some respondents faced discrimination in health facilities, including being refused care. Rather than access free government healthcare facilities, study participants opted for self-medication or paid services:

I do not go to the public hospital because nobody pays attention to us. They do not treat us properly and try to get rid of us. (Seema, 25 years, completed 4 years of schooling)

#### Context of sex work

High mobility for sex work due to police and/or community harassment and unsafe sex work settings increase HIV vulnerability. As narratives highlight, engaging in sex in unfamiliar locations elevated exposure to violence, which was difficult to challenge:

At new places we are scared. We feel clients will take us somewhere unknown and we don’t know what they will do with us. (Bela, 24 years, no schooling)

Both public and secluded sex work settings were described as unsafe, exposing sex workers to condom refusal, sexual and physical violence and HIV/STIs. Respondents faced constant police harassment at public sex work locations, which they were unable to address:

We take clients to dark areas for sex. If the police see us, they harass us. (Swara*,* 26 years, no schooling)

Meeting clients in private spaces also increased vulnerability to abuse, including rape. As highlighted below, after agreeing to have sex in a client’s home, the respondent was gang-raped and had little control over the situation:

Clients invite me to their room but I do not go. I am scared how they will behave there. Because some time ago, a client took me to his house and seven men had forced sex with me and paid nothing. (Reena, 21 years, completed 10 years of schooling)

### Coping with stigma and violence

Respondents described adopting a variety of coping strategies and using support structures at the micro, meso and macro levels.

#### Micro level: agency and resilience

Most often respondents tried to avoid harassment by running away from violence perpetrators, while some opted not to disclose their ‘stigmatised’ sex work profession:

My *guru* does not know about it [sex work]. I do it secretly. If he comes to know he will throw me out. (Ila, 32 years, no schooling)

Some respondents adopted more direct forms of resistance. One respondent regularly bribed the police to avoid harassment, while another confronted the police for discriminating against transgender persons and not registering a complaint:

I fought with my neighbour. He abused me for no reason. I filed a police complaint but they did not pay attention. I threatened the police saying, ‘If you do not take action, I will do something to myself and say it happened because of you. People behave like this with us because of you.’ Then the police arrested him and kept him in jail*.* (Seema, 25 years, completed 4 years of schooling)

Respondents also openly resisted pressure from *gurus* or abandoned violent and untrustworthy partners:

I left my boyfriend because he deceived me. I would give him my money to keep. He fell in love with a sex worker and started spending my money on her. When I got to know I abused him and drove him out. (Ila, 32 years, no schooling)

To challenge client violence and condomless sex, respondents negotiated condom use prior to sex, or insisted on advance payment so they could better negotiate condom use. Some refused clients who were not amenable to reason:

I immediately say, ‘If you don’t want to use a condom, go to another sex worker.’ Some fight but we try and convince them*.* (Alia, 20 years, completed 10 years of schooling)

Respondents also described avoiding unsafe public spaces where they were vulnerable to client violence:

My friend had a very bad experience. She had a regular client. One day he took her to a hotel and there were five more people there. That night six persons had sex with her. For this reason we do not go to hotels or lodges no matter how much a client pays. (Ravina, 24 years, no schooling)

While most respondents did not report violence to the police, one respondent described the protective role of the police in addressing client-perpetrated violence:

If a client threatens or beats me, I call the police. They will arrest him. A drunk client did not pay. He asked me for money and threatened to beat me. I called the police. The client paid me, then the police took him away. (Punita, 23 years, completed 7 years of schooling)

Less commonly reported was emotional support from biological families:

I went back to my village and convinced my family. They said they would help me whenever I needed. Since then I go to my village regularly. (Vandana, 20 years, no schooling)

#### Meso level: community networks

Peer support was the most commonly described coping resource. Peers were accepting of respondents’ transgender identity and helped them integrate into the new environment and cope with depression and personal stress. They also provided protection from daily violence*,* financial assistance and other support.

Some transgender persons saw me crying. I told them, ‘I like living like a girl, what is my mistake? I want to end my life now.’ One person said, ‘We are all like you. Have we been defeated in life? We also earn money and live. No one bothers us. In Mumbai we all stay together. Will you come with us?’ So I went with them to Mumbai. (Dia, 25 years, completed 9 years of schooling)

The *guru* supports us. If there is a fight, or police raid, the *guru* gets us released. Our *guru* also gives us money. When I am sick my transgender girlfriends look after me. (Sania, 30 years, completed 5 years of schooling)

A few narratives describe collective action with community networks to take control of abusive situations:

After sex, clients say, ‘I did not enjoy it, return my money,’ and fight with us. [In such cases] our *guru* and peers support us … all the sex workers come together and beat *thalis* [metal plates] and make a noise. The client is forced to run away. (Vinita, 28 years, completed 2 years of schooling)

#### Macro level: structural support

Some respondents described the supportive role of local NGOs, including advocacy with the police, bail for arrested transgender sex workers, linkages to health services and addressing violence:

Earlier we had trouble from the police and rowdies. But since the NGO started working here we do not face such problems. They speak to the police on our behalf. Harassment by rowdies has also reduced. The police also help us. (Ila, 32 years, no education)

## Discussion

This study in Maharashtra, India, confirms earlier evidence that transgender sex workers face pervasive stigma and violence, creating a high-risk environment for HIV transmission (Bockting et al. 2013; Sugano, Nemoto, and Operario 2006; Wilson et al. 2011). Due to multiple and reinforcing marginalised identities and social inequities (Logie et al. 2011) stemming from transgender identity, gender non-conforming behaviour, same-sex relations, sex work, transphobia, economic and housing insecurity, employment discrimination and poverty (Nemoto et al. 2014; Sugano, Nemoto, and Operario 2006; Wilson et al. 2011), respondents in our study are at elevated exposure to violence and poor health outcomes (Logie et al. 2011; White Hughto, Reisner, and Pachankis 2015). Stigma and violence are perpetrated within unequal power relationships and interlinked social and structural systems at the micro, meso and macro levels, legitimising marginalisation, discrimination and powerlessness (Parker and Aggleton 2003). HIV programmes must address the multi-level social-structural factors that elevate vulnerability and have implications for prevention and care.

While respondents in this study face extreme human rights violations, including physical violence, forced sex and rape, felt and internalised stigma (micro level) and the fear of violence undermine their ability to challenge perpetrators, report violence and negotiate condom use; notably, internalised stigma is associated with low self-worth, depression and anxiety, which are linked to HIV vulnerability, including physical and sexual violence, lack of social support and increased sexual risk taking among transgender persons (Bockting et al. 2013; Nemoto et al. 2004; Poteat et al. 2015). At the partnership level, respondents lack family support, which has been linked to isolation, low self-esteem, depression and lack of safety in public spaces among transgender persons elsewhere (Nemoto et al. 2014). In sexual transactions, power dynamics and clients’ control over the transaction process, fuelled by the perception that transgender sex workers are sexual objects whose rights need not be respected, create a context where violence and condomless sex are the norm. For devalued transgender sex workers seeking partnership stability, condomless intercourse helps establish trust and intimacy; however, along with direct exposure to HIV transmission, non-condom use in regular partnerships is associated with HIV vulnerability factors, including high rates of substance misuse and low self-esteem and self-efficacy among transgender persons (Nemoto et al. 2014). In our study, ‘regular’ partners were not willing to accept transgender sex workers’ stigmatised identity and occupation, increasing the likelihood of respondents engaging in sex with unfaithful and abusive partners (Nemoto et al. 2004).

In India, transgender persons’ visibility in public spaces for sex work and begging, as well as their inability to meet the feminine role of procreation, threaten gender-affirming norms, leading to pervasive stigma and violence (Kalra 2012). As described above, some community members (meso level) commit acts of extreme violence without fear of reprisal within a culture that marginalises and silences the voices of transgender persons and condones violence against gender non-conforming persons. Similar findings are reported among Indian *kothis* (feminised men who have sex with men), who experience elevated violence as a punishment for challenging traditional gender roles and as an affirmation of the perpetrator’s masculinity (Chakrapani et al. 2007).

Structural factors (macro level) reinforce stigma and violence and elevate HIV vulnerability. In this study, economic marginalisation was reported, increasing exposure to transphobia (Nemoto, Bödeker, and Iwamoto 2011), and discrimination in health settings compromised care seeking (Chakrapani et al. 2011). The criminalisation of homosexuality and respondents’ inability to seek redress allows the police to perpetuate violence with impunity; at the same time, police harassment diminishes transgender sex workers’ control over work spaces, client choice and condom use (Reza-Paul et al. 2012; Rhodes et al. 2008). Factors associated with sex work in our study – street-based solicitation (Dandona et al. 2005) mobility and migrant status (Ganju, Mahapatra, and Saggurti 2013; Ramesh et al. 2012; Saggurti et al. 2011) and early exposure to violence (Sugano, Nemoto, and Operario 2006) – are linked to HIV/STI vulnerability, inconsistent condom use, violence and alcohol consumption prior to sex. In coercive settings, transgender sex workers are unable to access social, legal and health rights and entitlements (Wilson et al. 2011).

Corroborating evidence of marginalised communities’ agency and resilience in settings of extreme violence (Logie and Daniel 2016), our study shows that respondents adopted ‘survival’ strategies to shift power relations and mobilise against abuse. Programmes could build on these demonstrated models of resilience to develop a collective response to protect sex workers’ rights.

While this study provides insights into transgender sex workers’ experiences of stigma and violence in the context of HIV vulnerability, its findings need to be considered in light of certain limitations. The study included a sample of purposively selected respondents, and hence findings may not be generalisable to other contexts. There may also have been recall bias, particularly when describing early experiences, a social desirability bias when reporting sensitive information, and some nuances may have been overlooked in translating interviews. As trained and experienced investigators conducted the interviews, we believe the study was able to minimise potential reporting biases and limit inconsistencies in translation. Finally, we note that this study was conducted in the early stages of the Avahan community mobilisation programme for marginalised groups, including transgender persons, in Maharashtra; however, the study results remain potentially relevant given that transgender persons continue to experience high levels of stigma and targeted violence in India (Shaw et al. 2012).

Despite these limitations, the study findings have important implications for HIV prevention. Community mobilisation is a promising vulnerability reduction strategy for sex workers and could be effective in safeguarding transgender sex workers’ rights and reducing HIV vulnerability. Collectivisation has been liked to higher autonomy and condom use negotiation, reduced violence and increased access to entitlements among sex workers in India (Gurnani et al. 2011; Saggurti et al. 2013). Community crisis response systems can provide immediate redress for violence incidents, and community drop-in centres can provide a safe violence-free meeting space to build collective resilience and reduce social isolation (White Hughto, Reisner, and Pachankis 2015).

At the individual level, transgender persons must be made aware of their rights, linked to community-based legal services, and supported to access social entitlements, financial inclusion schemes and employment opportunities other than sex work (Ganju et al. 2016). Interventions must provide transgender sex workers with HIV prevention information, including the need for consistent condom use in all partnerships. Additionally, transitioning transgender persons should be linked with adult transgender persons to learn coping strategies, and families should be sensitised to the importance of accepting transgender persons’ gender status.

To build supportive norms and challenge transgender-related stigma (meso), community leaders, *gurus* and peers must create awareness of transgender persons’ rights and encourage community members to integrate this marginalised group into society. Media campaigns that share transgender persons’ stories of hopelessness, recognition, support, love and resilience could help promote community acceptance and address discrimination (White Hughto, Reisner, and Pachankis 2015).

In the context of pervasive police violence and human rights abuses, the police and key stakeholders should be made aware of transgender persons’ rights and the need for immediate violence redress to reduce vulnerability. Health providers must be trained to provide non-judgmental services and sensitised to screen all transgender persons for mental health status and outcomes of sexual and physical violence, and provide necessary services. The use of trained transgender persons as counsellors in health centres could help address discrimination based on multiple stigma (Poteat et al. 2015).

## Conclusion

Our study findings show that transgender sex workers in Maharashtra, India, experience pervasive stigma and violence due to multiple marginalised social identities, which reinforce and intersect with social inequities, at the micro, meso and macro levels, fuelling HIV vulnerability. Respondents adopted ‘survival’ strategies to shift power relations and mobilise against abuse. Community mobilisation interventions offer a promising vulnerability reduction strategy for sex workers, and could be effective in safeguarding transgender sex workers’ rights and reducing HIV-related vulnerability.

## Disclosure statement

No potential conflict of interest was reported by the authors.

## Funding

This paper was funded by a grant to the Population Council from the Bill & Melinda Gates Foundation through Avahan, the India AIDS Initiative. The views expressed herein are those of the authors and do not necessarily reflect the official policy or position of the Bill & Melinda Gates Foundation or Avahan.
